# Molecular Epidemiology and Genetic Characterization of PCV2 Circulating in Wild Boars in Southwestern Ethiopia

**DOI:** 10.1155/2022/5185247

**Published:** 2022-09-28

**Authors:** Dechassa Tegegne, Girma Tsegaye, Sultan Aman, Giulia Faustini, Giovanni Franzo

**Affiliations:** ^1^School of Veterinary Medicine, Jimma University College of Agriculture and Veterinary Medicine, P.O. Box 307, Jimma, Ethiopia; ^2^Department of Animal Medicine, Production and Health (MAPS), University of Padua, Legnaro (PD), Padua, Italy

## Abstract

Porcine circovirus type 2 (PCV2) is one of the most relevant infectious agents affecting domestic pigs. Recently, a surprising PCV2 genetic heterogenicity has been reported in Africa. Nevertheless, the knowledge of the epidemiology of PCV2 in African countries, in both domestic and wild species, is limited and sparse. Having this in mind, in the present study, the PCV2 circulation and its molecular epidemiology in Southwestern Ethiopia have been investigated by collecting 64 samples from domestic pigs, wild boars, and warthogs. PCV2 genome presence was detected and quantified using qPCR and ORF2 sequencing was attempted on positive samples. Ten samples, 8 wild boars, 1 domestic pig, and 1 warthog, tested PCV2 positive. Complete ORF2 sequences were obtained from 5 wild boars; 4 of those were classified as PCV2d and 1 as PCV2b. Both PCV2b and PCV2d were related to strains of Asian origin, most commonly from China. The role of this country in the exportation of PCV2 strains in Ethiopia, and Africa in general, might be supported by the crescent economic relationship between the two continents. The obtained evidence also testifies to the inadequacy and/or poor application of biosecurity measures separating wild and domestic animals. Further, extensive and systematic studies should be performed to more deeply characterize the molecular epidemiology of PCV2 in this region, in order to improve our understanding of these ecological niches in the evolution and dispersal of PCV2.

## 1. Introduction

Porcine circovirus type 2 (PCV2) is one of the most relevant infectious agents affecting domestic pigs. It has been associated with several clinical conditions, collectively named porcine circovirus diseases (PCVD), ranging from systemic disease (PCV2-SD), which includes what was initially described as PCV2-associated pneumonia and PCV2-associated enteritis, reproductive disorders (PCV2-RD), and porcine dermatitis and nephropathy syndrome (PDNS). Subclinical infection (PCV2-SI) is also considered part of PCVD since, although not featured by overt clinical signs, it leads to a decrease in productive performance and often represents a source of economic losses for the farmer, even higher than the clinical forms [1–4].

PCVD is a typical example of a multifactorial disease, requiring the interaction among the pathogen, host, and environment to hesitate in clinical or productive effects. The change and intensification in the swine production system, typical of modern farming in high-income countries, clearly played a pivotal role [5]. In fact, despite PCV2 being identified as a swine pathogen in the late 90s, retrospective studies and analysis based on the molecular clock revealed a much more ancient origin and undetected circulation [6]. Similarly, different studies have revealed PCV2 infection in wild boars and other wild animals, although negligible evidence of clinical signs has been provided in these species [7–13]. Also, extensively raised pigs appeared less affected. Such a scenario well conforms to the African one, where, although extremely limited, the first epidemiological data have started to accumulate. Even if PCV2 economic impact may not be considered a priority in African countries, the same cannot be said for their epidemiological, evolutionary, and ecological potential.

PCV2 is a single-stranded DNA virus (ssDNA) with a circular genome of about 1.76 kb. Similarly to other ssDNA viruses, it is characterized by a high mutation (i.e., 10^−3^–10^−4^ substitution/site/year) and recombination rate, leading to remarkable genetic and phenotypic variability [6, 14, 15]. Although several open reading frames (ORFs) have been predicted *in silico*, just a few have been properly characterized; ORF1 and ORF2 in particular have been more extensively investigated. ORF1 encodes 2 proteins (Rep and Rep') involved in rolling circle replication, while ORF2 encodes Cap, the only constituent of the viral capsid, that is involved in the viral attachment and is the main target of host immune response [14]. Because of ORF2 higher genetic heterogenicity, it is commonly sequenced for molecular epidemiology studies and genotyping. Currently, 9 genotypes have been formally identified, although just 3 of those (i.e. in ascending order, PCV2 a, b, and d) are the most prevalent and widespread [15, 16]. Nevertheless, minor genotypes have been identified in unexpected countries and ecological niches. The detection of PCV2c, previously considered extinct, in feral pigs in the Brazilian region of Pantanal and in Namibian warthogs is probably the most obvious example [17, 18]. Also, PCV2g has been reported in African pigs [10]. Therefore, the capability of these often-neglected species and countries to host minor or even currently undetected genotypes and genetic variants appears clear, and they could affect and contribute to PCV2 evolution. This scenario could represent a threat to the swine industry at the global level, leading to the emergence of new variants with unpredictable behavior. Based on these considerations, in the present study, PCV2 circulation and its molecular epidemiology have been investigated, for the first time, in domestic pigs, wild boars (*Sus scrofa*), and warthogs (*Phacochoerus africanus*) in Southwestern Ethiopia.

## 2. Material and Methods

Sixty-four samples were collected from domestic pigs, wild boars, and warthogs from September to December 2021 in Oromia regional state. Samples were represented by tissue from wild boars, while feces were collected from domestic pigs and warthogs. All domestic pigs' samples were collected from the intensively managed farm of Jimma University, College of Agriculture and Veterinary Medicine (JUCAVM) in Southwestern Ethiopia. None of the herds reported health problems.

Samples from free-living adult wild boars were collected from the Buno Bedele zone in Dega and Dabo Hana districts in Southwestern Ethiopia from freshly shouted wild boars by local hunters for human consumption or by farmers to protect their cropland. The post-mortem examination was carried out in the nearby regional veterinary clinic laboratory. The liver, kidney, heart, spleen, and lung were collected after an aseptic incision and applied to the Whatman® FTA® card technology (WhatmanTM) and allowed to dry for 30 minutes at room temperature.

In the Dinsho national park, Bale zone, Southwestern, Ethiopia, healthy warthog's fresh feces samples (∼ 10 g) were collected from the top of a pile of freshly passed feces. Stool suspensions (10%) were made in PBS pH 7.2 and centrifuged at 8000 × *g* for 5 min. 125 *µ*L of each of the obtained solutions were applied to one of the four FTA card sample areas and dried for 30 minutes at room temperature. The FTA cards were delivered to the laboratory of infectious disease of the dept. MAPS, Padua University, for PCV2 infection diagnosis and molecular characterization.

From each of the 4 FTA card sample areas, a strip of 1 cm (height) × 0.3 mm (width) was trimmed. The 4 obtained strips were pooled and mixed in 500 *µ*L of PBS and incubated at room temperature for 1 h while continuous vortexing. A volume of 100 *µ*L of the supernatant was extracted using the kit Viral DNA/RNA (A&A Biotechnology) according to manufacturer instructions. An exogenous internal control, provided by the QuantiNova Pathogen+ IC kit (Qiagen), was added before extraction.

The PCV2 genome presence was detected and quantified using the qPCR assay described in Franzo et al., [11]. Complete ORF2 amplification and sequencing were attempted on all positive samples following the approach described by Franzo et al., [11]. Chromatogram quality was evaluated by visual inspection in FinchTV (https://www.geospiza.com) and consensus sequences were generated using ChromasPro (ChromasPro Version 2.0.0, Technelysium Pty Ltd). The occurrence of double peaks, which could be suggestive of co-infections with multiple genotypes, was assessed both by visual inspection and using the dedicated software Poly Peak Parser [19]. Finally, strains were classified into genotypes by comparison with the set of reference sequences proposed by Franzo and Segales [15]. In more detail, the obtained sequences were aligned at codon level using the mafft algorithm implemented in TranslatorX [20] and a phylogenetic tree was reconstructed using MEGA X [21].

Finally, to preliminary assess the relationship with other countries, a complete dataset of more than seven thousand and five hundred ORF2 sequences with known collection countries were downloaded from GenBank and aligned with the reference and Ethiopian ones. Poorly aligned reads, those with premature stop codons or unknown bases, were removed from the alignment. Thereafter, a phylogenetic tree was reconstructed using FastTree2 [22].

## 3. Results

**Figure 1:**
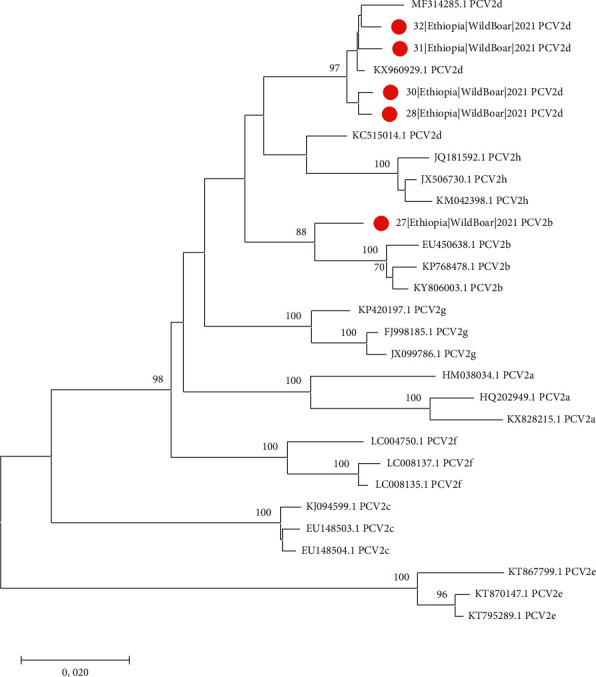
Neighbor-joining phylogenetic tree depicting the relationship between strains sequenced in the present study and the set of reference sequences suggested by Franzo and Segales [15]. The bootstrap support, when higher than 70, is reported nearby the corresponding node.

Sixty-four samples were collected from *Suidae* located in Oromia regional state; in brief, 40 samples were collected from wild boars (*n* = 40) from Buno Bedele and 8 samples (*n* = 8) were collected from domestic pigs from the intensive farm of Jimma University, College of Agriculture and Veterinary Medicine in Southwestern Ethiopia; 16 samples were collected from warthogs from Bale zone, Dinsho park in Southwestern Ethiopia. All animals were in good clinical condition, and no macroscopic lesions were detected in wild boars. Ten samples tested PCV2 positive (Table 1), although at a low titer (i.e. 100–10 genome copies/mL of starting material). Eight of them were wild boars, one was represented by a pool of domestic pigs and 1 originated from a warthog. Complete ORF2 sequences could be obtained from 5 wild boars only (Acc. Number ON337865-ON337869). Comparison with reference sequences allowed us to classify 1 of those (i.e. strain 27), sampled in the Dega region, as PCV2b, while the remaining 4 (i.e. strains 28, 30, 31, and 32), originating from the Dabo area, were PCV2d (Figure 1). Comparison with the international dataset revealed the PCV2b strain to be part of a clade including European and Asian strains. PCV2d sequences, featured by a certain variability among them (i.e., mean *p*-distance: 0.01; range 0.005–0.014) were part of different clades. However, in all instances, they demonstrated a close relationship with Chinese sequences (Supplementary Figure 1).

## 4. Discussion

The present study reports the detection of PCV2 in Ethiopian domestic pigs, wild boars, and warthogs as well, confirming the broad distribution and circulation of this virus in the African continent. In the area where samples were collected, contacts between wild boars and warthogs occur, while strain exchange with domestic rural breeds is unlikely due to the absence of pig production due to religious factors. Therefore, a recent or frequent introduction from domestic populations can likely be excluded. All collected samples originated from apparently healthy subjects and displayed a low viral titer despite the fact that the animals were not vaccinated against PCV2. Likely, the wildlife (i.e., wild boar and warthogs) or the less intensive farming condition compared to high-income countries removed most of the co-factors that are typically required for high viral replication and clinical sign development. If pathogen-related factors, e.g. a lower virulence, were also involved remains to be established. A previous study reported the detection of PCV2a and PCV2b genotypes in Ethiopia [10]; unfortunately, the low viral titer hindered the sequencing of all positive samples. In one of the sequenced samples, the presence of PCV2b was confirmed, while the other 4 sequences were classified as PCV2d, further demonstrating the breadth of genetic heterogeneity featuring Ethiopian molecular epidemiology. A geographic pattern was highlighted in the present study, with different genotypes being identified in different areas. Nevertheless, due to the limited sequence number, it is impossible to quantify the effect of chance in the observed scenario.

Since the previous sequences originated from samples collected in 2014, it could be speculated that the higher PCV2d frequency might reflect the worldwide genotype shift [6]. However, also in this case, the effect of chance or geographical clustering cannot be excluded, and more extensive and systematic studies should be performed.

Warthogs were also confirmed to be susceptible to the infection. In a recent Namibian study, a surprisingly high frequency of PCV2c was proven in this species [18]. If a host preference/association is in place or if the observed scenario was due to local peculiarities is currently unknown since the molecular characterization and genotyping of the detected strain were not possible in the present study.

The comparison of the obtained sequences with a broad international dataset revealed a quite complex scenario. The PCV2b strain clustered with strains reported in Europe and Asia. The wide spread and dispersal of this genotype at the global level impede the identification of the precise source of introduction.

However, a previous study estimated a likely PCV2b introduction in Ethiopia from Asia [10]. Therefore, a similar importation pathway could be expected. On the other hand, Ethiopian PCV2d strains were part of different clusters, thus suggesting multiple independent introduction events. Remarkably, in all instances, closely related strains were of Asian origin, more commonly from China. The role of this country in the exportation of PCV2 strains in Ethiopia, and in Africa in general, appears likely and is supported by the growing economic relationship between the two continents [23]. Nevertheless, such a strain exchange is expected to affect commercial pigs rather than wild ones. How the passage occurred remains to be established. Regardless of this knowledge gap, the obtained evidence testifies to the inadequacy and/or poor application of biosecurity measures separating wild and domestic animals. Therefore, further efforts should be made in this sense to protect both commercial pigs from strains of wild origin, and to prevent the spread of the infection into wildlife.

Overall, the present study confirms the relevant PCV2 infection frequency in different suid hosts in Africa, both domestic and wild ones, and further extends the knowledge of the genetic variability featuring Ethiopia, where all major PCV2 genotypes have been detected. A pivotal role of foreign strain importation, mainly from Asia, has also been proven and should suggest the implementation of more effective control measures to limit such a phenomenon. Furthermore, extensive and systematic studies should be performed to more deeply characterize the molecular epidemiology of PCV2 in this region and improve our understanding of these ecological niches in the evolution and dispersal of PCV2.

## Figures and Tables

**Table 1:** Positive samples distribution.

Pool (4 samples) number	Code in pool	Districts	Species	Production type	Sex	Age^*∗*^	Sample type	Health status	PCV2
24	P1a	Dabo hana	Wild boar	Wild	M	Adult	Liver	Health	Low titter
	P1b	Dabo hana	Wild boar	Wild	M	Adult	Liver	Health	Low titter
	P1c	Dabo hana	Wild boar	Wild	M	Adult	Liver	Health	Low titter
	P1d	Dabo hana	Wild boar	Wild	M	Adult	Liver	Health	Low titter

25	P2	Dabo hana	Wild boar	Wild	F	Adult	Liver	Health	Low titter
	P3a	Dabo hana	Wild boar	Wild	F	Young	Liver	Health	Low titter
	P3b	Dabo hana	Wild boar	Wild	F	Young	Spleen	Health	Low titter
	P5e	Dabo hana	Wild boar	Wild	F	Young	Liver	Health	Low titter

26	P4a	Dabo hana	Wild boar	Wild	M	Adult	Spleen	Health	Low titter
	P4b	Dabo hana	Wild boar	Wild	M	Adult	Heart	Health	Low titter
	P4c	Dabo hana	Wild boar	Wild	M	Adult	Kidney	Health	Low titter
	P7e	Dabo hana	Wild boar	Wild	M	Adult	Lung	Health	Low titter

27	P5a	Dega	Wild boar	Wild	F	Adult	Kidney	Health	Complete ORF2 sequenced
	P5b	Dega	Wild boar	Wild	F	Adult	Heart	Health	Complete ORF2 sequenced
	p5c	Dega	Wild boar	Wild	F	Adult	Spleen	Health	Complete ORF2 sequenced
	P5d	Dega	Wild boar	Wild	F	Adult	Liver	Health	Complete ORF2 sequenced

28	P7a	Dabo hana	Wild boar	Wild	F	Adult	Lung	Health	Complete ORF2 sequenced
	P7b	Dabo hana	Wild boar	Wild	F	Adult	Heart	Health	Complete ORF2 sequenced
	P7c	Dabo hana	Wild boar	Wild	F	Adult	Kidney	Health	Complete ORF2 sequenced
	P7d	Dabo hana	Wild boar	Wild	F	Adult	Liver	Health	Complete ORF2 sequenced

30	P9a	Dabo hana	Wild boar	Wild	M	Adult	Liver	Health	Complete ORF2 sequenced
	p9b	Dabo hana	Wild boar	Wild	M	Adult	Spleen	Health	Complete ORF2 sequenced
	p9c	Dabo hana	Wild boar	Wild	M	Adult	Kidney	Health	Complete ORF2 sequenced
	p9d	Dabo hana	Wild boar	Wild	M	Adult	Heart	Health	Complete ORF2 sequenced

31	p10a	Dabo hana	Wild boar	Wild	M	Adult	Liver	Health	Complete ORF2 sequenced
	p10b	Dabo hana	Wild boar	Wild	M	Adult	Heart	Health	Complete ORF2 sequenced
	p10c	Dabo hana	Wild boar	Wild	M	Adult	Kidney	Health	Complete ORF2 sequenced
	p10 d	Dabo hana	Wild boar	Wild	M	Adult	Spleen	Health	Complete ORF2 sequenced

32	p11a	Dabo hana	Wild boar	Wild	M	Adult	Spleen	Health	Complete ORF2 sequenced
	p11b	Dabo hana	Wild boar	Wild	M	Adult	Liver	Health	Complete ORF2 sequenced
	p11c	Dabo hana	Wild boar	Wild	M	Adult	Heart	Health	Complete ORF2 sequenced
	p11 d	Dabo hana	Wild boar	Wild	M	Adult	Kidney	Health	Complete ORF2 sequenced

35	P16	Dabo hana	Pig	Domestic	F	Adult	Feces	Health	Low titter
	P17	Dabo hana	Pig	Domestic	M	Adult	Feces	Health	Low titter
	P18	Dabo hana	Pig	Domestic	F	Young	Feces	Health	Low titter
	P19	Dabo hana	Pig	Domestic	F	Adult	Feces	Health	Low titter

37	B1	Dinsho	Warthog	Wild	M	Adult	Feces	Health	Low titter
	B2	Dinsho	Warthog	Wild	M	Adult	Feces	Health	Low titter
	B3	Dinsho	Warthog	Wild	F	Adult	Feces	Health	Low titter
	B4	Dinsho	Warthog	Wild	F	Adult	Feces	Health	Low titter

## Data Availability

The datasets generated and analysed during the current study are available in the GenBank repository (Acc. Number: ON337865-ON337869).

## Supplementary Materials

Supplementary Materials Supplementary Figure 1. Maximum likelihood phylogenetic tree depicting the relationship between strains sequenced in the present study and a collection of ORF2 sequences of PCV2 sequences collected at the global level.
