# Supplementary figures and images for: Molecular Epidemiology and Genetic Characterization of PCV2 Circulating in Wild Boars in Southwestern Ethiopia
Source: J Trop Med. 2022 Sep 28;2022:5185247. doi: 10.1155/2022/5185247 (PMC9534679; doi:10.1155/2022/5185247)

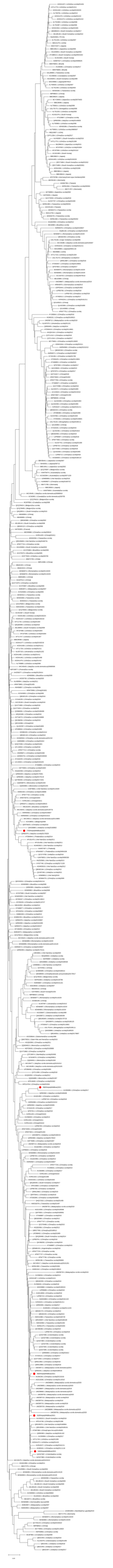

Supplement: Supplementary Materials — Supplementary Figure 1. Maximum likelihood phylogenetic tree depicting the relationship between strains sequenced in the present study and a collection of ORF2 sequences of PCV2 sequences collected at the global level. [file 5185247.f1.pdf]
